# Is there a link between peripheral inflammation and blood brain barrier integrity in children with attention-deficit/hyperactivity disorder? A case-control study

**DOI:** 10.1186/s12887-024-05254-4

**Published:** 2024-11-26

**Authors:** Samira Zien Sayed, Zienab Osama Hassan, Wedad M. Abdelraheem, Rasha Samir Refaat, Ibtehal Saad Abuelela

**Affiliations:** 1 Department of Pediatrics, Faculty of Medicine, Minia University, El-Minia, Egypt; 2 Department of Medical Microbiology and Immunology, Faculty of Medicine, Minia University, El-Minia, Egypt; 3 Department of Neuropsychiatry, Faculty of Medicine, Minia University, El-Minia, Egypt

**Keywords:** ADHD, Blood brain barrier, Claudin-5, Peripheral inflammation, Neutrophil/lymphocyte ratio and children

## Abstract

**Background:**

Claudin-5 is a vital constituent of tight junctions, which are critical elements of the blood-brain barrier. In people with neuropsychiatric disorders, peripheral inflammation is often found, although it is less common in healthy populations. The objective of this study was to examine the relationship between Claudin-5, peripheral immune cells, and the severity of symptoms in children with attention deficit hyperactivity disorder (ADHD).

**Methods:**

The study included a cohort of 33 children diagnosed with ADHD and 29 control subjects, all aged between 5 and 12 years. The intensity of ADHD symptoms was evaluated using Conner’s questionnaire, which the parents completed. Each kid had serum level measurements of Claudin-5 and a complete blood count in order to establish a correlation with symptoms of ADHD.

**Results:**

Serum Claudin-5 levels are lower in the ADHD group compared to the control group; median (IQR) = 30.94 (4-137) and 44.12 (4–223.3) respectively (*p* = 0.69). The levels of neutrophils and neutrophil/lymphocyte ratio are significantly higher in ADHD than in controls (*p* = 0.011 and 0.015, respectively). Lymphocytes have a significant positive correlation with ADHD symptoms severity, namely, total Conner’s scale and inattention (*p* = 0.021 and 0.004 respectively), while NLR has a significant negative correlation with total Conner’s score and impulsivity (*p* = 0.046, *p* = 0.038), also a negative correlation yet not significant between serum Claudin-5 level and total Conner’s score, hyperactivity, impulsivity, and inattention. Neutrophils were found to have a significant positive linear regression with Claudin-5 (*p* = 0.023).

**Conclusion:**

These results revealed that BBB integrity is affected in ADHD children, as claudin-5 levels were found to be lower in children with ADHD, lymphocytes were found to be associated with increased ADHD symptoms severity, and NLR was associated with decreased symptoms severity, which may be via the positive effects of increased neutrophils on Claudin-5 levels.

## Background

Attention deficit hyperactivity disorder (ADHD) is a disorder of brain development marked by inattention and hyperactivity-impulsivity, which hinders a person’s capabilities [1]. Whereas it has been proposed that inherited, neurological, and socioeconomic factors influence the root cause of ADHD, its etiology remains uncertain [2]. Research studies on the physiological variances in ADHD have found many differences related to structure [3], function [4], and biochemical [5] distinctions among ADHD and non-ADHD participants.

The blood-brain barrier (BBB) integrity is of concern, and recent researches have shown that it may play a role in the pathophysiology of various psychiatric diseases like autism, personality disorders and mood disorders [6]. In a similar way, the association between ADHD and BBB was investigated in earlier research, finding modifications in proteins associated with tight junctions especially claudin-5 [7, 8]. Claudin-5 is a molecule that is currently being studied in numerous diseases, including disorders of mental health [9].

Claudin-5 is an essential part of capillary tight junctions in the BBB, and Claudin-5 malfunction increases the brain’s exposure to hazardous environmental variables [10]. Claudin-5 has been linked to mental illnesses such as personality disorders as bipolar disorder, psychosis, and obsessive compulsive disorder [9, 11–13]. Also, Furthermore, it has been proven that immunologic and inflammatory mechanisms may play an important role in the pathophysiology and establishment of neurological mental illnesses such as ADHD [14]. Many factors might contribute to this, including alterations in inflammatory mediators, healthy microglia, chemical mediators, astrocytes, and the effect of oxidative stress [15].

Many studies found youngsters with ADHD had substantially more NLR, PLR, and circulating neutrophil levels than the children in the normal control group [16, 17]. As a result, blood biomarkers associated with inflammation may aid in figuring out the illness status and may anticipate the eventual outcome of children with ADHD in a variety of therapeutic settings [16, 18].

The aim of this study is to ascertain the presence of affected BBB integrity via comparing the levels of Claudin-5 between ADHD children and healthy controls, and to assess the role of inflammatory cells in the interpretation of ADHD symptoms. Also, to find out any association between these inflammatory cells and BBB integrity in children with ADHD in comparison to healthy controls.

## Patients and methods

### Study design and participants

The study group was composed of children diagnosed with ADHD between the ages of 5 and 12, without distinction to the gender, collected from individuals who were visiting the Pediatric Psychiatric Clinic of Minia University Hospital, Egypt, between January 2023 and October 2023. 56 eligible patients were recruited for the trial; 15 declined to participate, and 8 were ineligible due to meeting at least one of the criteria for exclusion. A total of 33 participants had been recruited for the research work. The inclusion criteria were (1) children diagnosed with ADHD according to DSM-5 criteria (2) drug naïve patients (no history of psychotropic medication administration), (3) aged from 5 to 12 years from both sexes, (4) only children whom care givers gave informed consent were included in the study.

A list of criteria for exclusion was used for the study group: Being a lifelong physical, metabolic, syndromic or neurologic condition; having a past record of head trauma; being diagnosed with any psychiatric disease other than ADHD and having previously used ADHD or any other type of psychotropic drug. We also eliminated children who had an acute or long-term infection that could raise blood indicators of inflammation or were taking drugs that could alter leucocyte and platelet numbers or performance.

The control group was made up of families of Minia University Hospital staff members. They were socio-demographically matched with ADHD patients who volunteered to participate and their guardians. The participants were assessed by expert psychiatrist to exclude the presence of any psychiatric illness. Those with no present or past behavioral disorders and no psychotropic medication usage were assigned to the control group, which included 29 children.

### Ethical consideration

Before gathering information, the project was approved by the Institutional Review Board (IRB), Faculty of Medicine, Minia University, under approval number (577:2023) dated 1 January 2023. The researcher got informed consent from the children’s parents/guardians after advising them of the study’s purpose and ensuring that the information they provided would stay confidential and used only for scientific purposes.

### Data collection

The medical evaluation encompassed the following areas: prenatal, natal, and postnatal time periods, including antepartum hemorrhage, infections, any maternal difficulties during the delivery process, cyanosis or jaundice in the neonatal period or being with low or high gestational weight. Then specific motor and mental milestones in development were evaluated. Anthropometric measures were obtained while wearing light clothes and without footwear.

### ADHD assessment

Parents of study participants were directed to experienced psychologists to evaluate the extent of ADHD symptoms using the Conner’s’ Parent Rating Scale Revised-Long edition [19]. The reliability and validity of the Arabic version used in this research work was tested by El-Sheikh et al. [20].

The Conner’s test evaluates individuals’ abilities in the domains of inattention, impulsivity, and hyperactivity. It comprises eighty questions that are graded on 14 subscales of symptoms of the disorder, and the average time required for administration is 25 to 30 min. It assesses the parents’ description of how their kid behaved during the last month on a 4-point reply scale. (0) Not true at all, (never or seldom), (1) just a little true (occasionally), (2) pretty much true (often, quite a bit), and (3) very much true (very often, very frequent) [20, 21].

### Venous sampling of the participants

#### Complete blood count (CBC)

Blood samples were taken in the morning, while the participants were fasting via a vein puncture into a test tube containing ethylenediaminetetraacetic acid (EDTA). The CBC was then tested with an automated cell counter analyzer. NLR was calculated by dividing the overall neutrophil number by the overall number of lymphocytes (average reference was: 1.2–4.4) PLR was computed by dividing the number of platelets by the number of lymphocytes (average value reference was: 75–199) [22].

#### Claudin-5 serum level assessment

Further venous blood samples from the case and control groups were taken in the morning and while the participants were fasting. The specimens of blood were centrifuged, and the separated serum samples were frozen at − 80 ℃ until used. Serum levels of claudin-5 were measured using commercial enzyme-linked immunosorbent assay (ELISA) kits (E-EL-H1630-Elabscience, Wuhan, China) and the determination range was (20-4500 ng/L) according to the manufacturer’s instructions. The degree of color change was measured utilizing the Biorad Microplate wavelength reader xMark (Bio-rad Laboratories, California, USA) system. All results were estimated as “ng/L” based on spectrophotometer-concentration calibrating graphs.

### Statistical analysis

Statistical analyses were carried out using SPSS 20.0 software (SPSS Inc., Chicago, IL, USA). The Kolmogorov-Smirnov test was used to ensure that all variables had normal distributions. Age, biochemical parameter levels, and scale scores were compared between the study and control groups using Mann-Whitney test and expressed by median and interquartile range (IQR) or Student’s t-tests expressed in mean ± standard deviation (SD), depending on distribution features. In addition, following the comparative analyses, Pearson or Spearman correlation analyses were used in the study group to investigate the correlations between biochemical markers and clinical factors. Finally, a linear regression model was run on the study group to see if Conner’s scale scores had incremental predictive value in predicting the Claudin-5 under investigation.

## Results

### Sample description

The study involved 33 children with ADHD (23 boys, 6 girls) and 29 control children (21 boys, 8 girls), with no significant difference between age and sex between ADHD children and the control group (Table 1).

As regards BMI, no significant difference was noticed between the case and control groups (*p* = 0.544). No significant difference regarding gestational age and breastfeeding (*p* = 0.142 and 0.318, respectively). ADHD children were noticed to have higher rates of birth via C.S. but still with no significant difference (*p* = 0.568). Children with ADHD had a more significant history of developmental delay than the control group (*p* = 0.02). It is noticed that children with ADHD had a significantly positive family history of one parent’s psychiatric illness than the control group (*p* = 0.010; Table 1).

### Inflammatory markers and serum Claudin-5

**Fig. 1:**
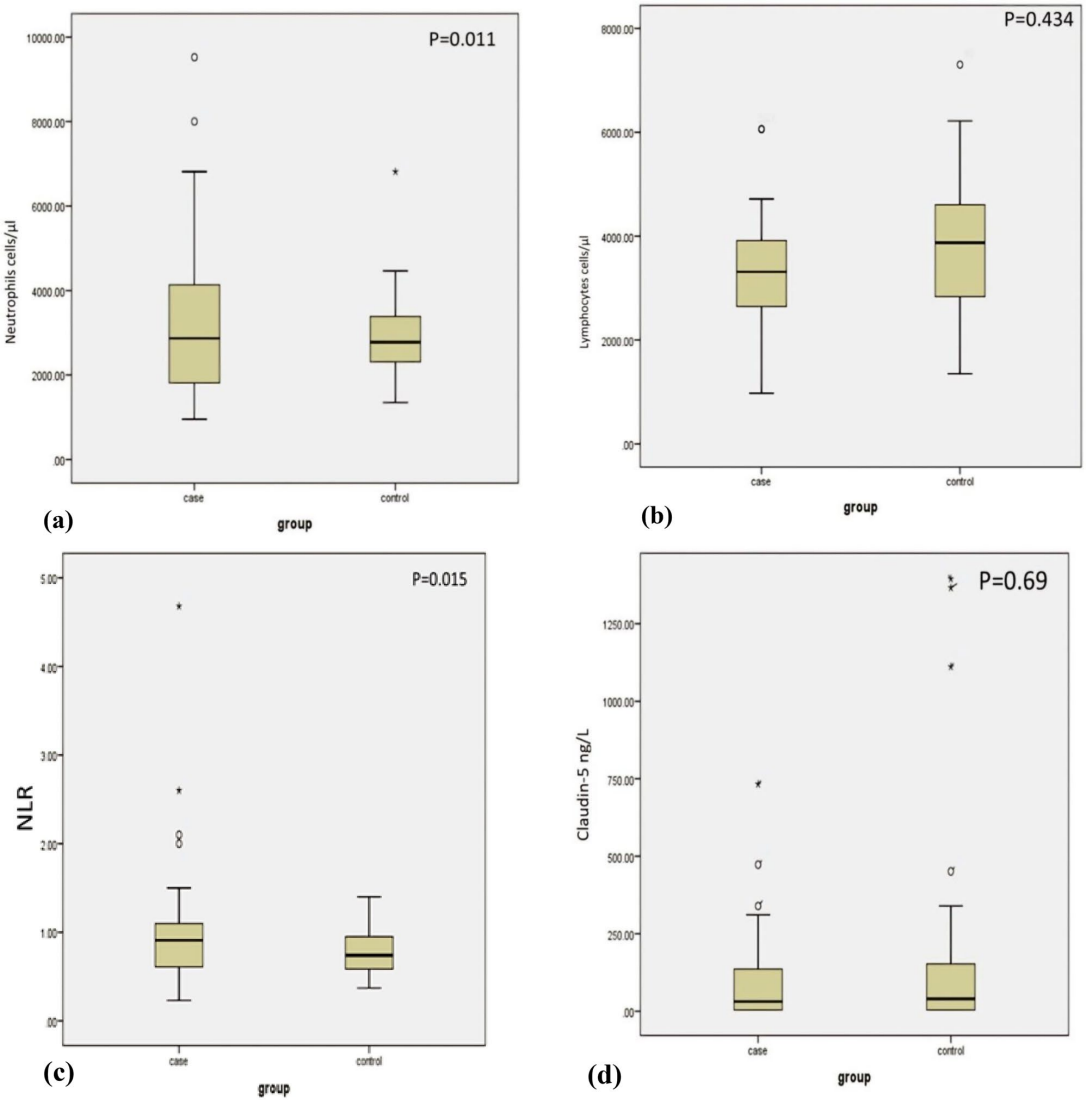
Box plots describe the distribution of variables; (**a**) Neutrophils, (**b**) Lymphcytes, (**c**) NLR and (**d**) Claudin-5 levels between ADHD and healthy children

As shown in Table 2; Fig. 1, despite there being no significant difference in the white blood cell (WBC) count between both groups, there is still a significant difference in the neutrophil counts, with higher levels in the ADHD group (*p* = 0.011). Also, NLR is significantly higher in cases than controls (*p* = 0.015). Lymphocytes show lower values in cases than controls, but yet an insignificant difference. Serum Claudin-5 levels were found to be abnormally distributed between cases and controls, so we used Mann Whitney test to compare medians between the 2 groups, and we found that Claudin-5 values (median and IQR) were lower in cases than controls; median (IQR) = 30.94 (4-137) and 44.12 (4–223.3) respectively, yet insignificant (*p* = 0.69).

A significant positive correlation was observed between WBCs and lymphocytes with Conner’s scale and the inattention subscale. However, a negative, significant correlation was observed between NLR and PLR with Conner’s scale, impulsivity, and inattention subscales. Regarding the correlation of symptom severity and Claudin-5 serum levels, a negative correlation was found between Claudin-5 and Conner’s total score, impulsivity, hyperactivity, and inattention scores, but this correlation is still insignificant (Table 3).

**Fig. 2:**
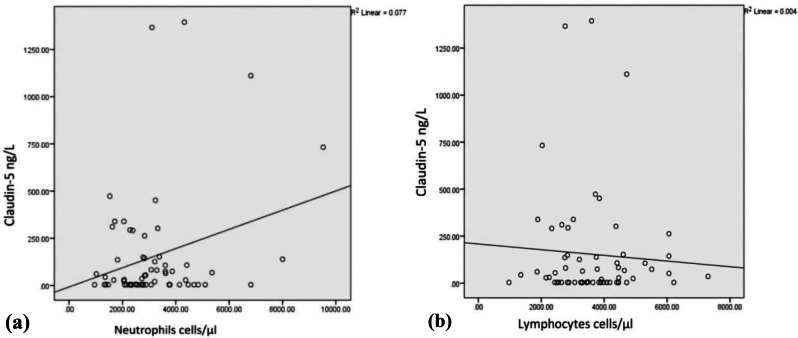
Correlation analysis between Claudin-5 with (**a**) Neutrophils and (**b**) Lymphoctes

On trying to analyze the interplay between Claudin-5 and inflammatory markers, a significant positive association was found between Claudin-5 and neutrophils rather than NLR (*p* = 0.023 and 0.558, respectively); however, a fair negative correlation was observed with lymphocytes (*p* = 0.154) (Table 4; Fig. 2).

**Table 1:** Demographic characteristics of ADHD and control groups

Variables		ADHD (NO = 33)	Control (NO = 29)	*P* value
Age (Years)	Mean ± SD^a^	8.24 ± 2.24	7.9 ± 2.57	0.397
Sex				
Boys	(N%)^b^	27(81.8%)	21(72.4%)	0.583
Girls	(N%)^b^	6(18.2%)	8(27.6%)	
Height (Cm)	Mean ± SD^a^	123.41 ± 41.95	118.53 ± 15.95	0.758
Weight (Kg)	Mean ± SD^a^	30.63 ± 10.71	29.68 ± 11.75	0.274
BMI (M^2^)	Mean ± SD^a^	19.99 ± 3.27	20.06 ± 3.30	0.544
GA (Weeks)	Mean ± SD^a^	37.45 ± 1.39	37.55 ± 1.20	0.142
Normal vaginal delivery	(N%)^b^	12(36.4%)	13(44.8%)	0.568
Breast feeding	(N%)^b^	23(69.7%)	19(65.5%)	0.318
Developmental delay	(N%)^b^	8(24.2%)	1 (3.4%)	0.020*
Father psychiatric illness	(N%)^b^	11(33.3%)	1 (3.4%)	0.010*
Father smoking	(N%)^b^	16(48.5%)	17(58.6%)	0.416
Screen media (hours/day)	Mean ± SD^a^	2.02 ± 2.30	1.91 ± 1.22	0.059

^a^ independent sample t test

^b^ chi-square test

*: Significant difference comparing between both groups at P value < 0.05

BMI; body mass index and, GA; gestational age, SD; standard deviation, N%; number and percent

**Table 2:** Comparison between Claudin-5 and peripheral inflammatory parameters between children with ADHD and control groups

Variables		ADHD (NO = 33)	Control (NO = 29)	*P* value
WBCs 10^3^cells/µl	Mean ± SD^a^	7.91 ± 2.85	7.75 ± 2.20	0.156
Neutrophils 10^3^cells/µl	Mean ± SD^a^	3.31 ± 1.98	2.98 ± 1.06	0.011*
Lymphocytes 10^3^cells/µl	Mean ± SD^a^	3.38 ± 1.11	3.89 ± 1.32	0.434
Platelets 10^3^cells/µl	Mean ± SD^a^	379.88 ± 145.14	353.82 ± 102.80	0.952*
NLR	Mean ± SD^a^	1.054 ± 0.83	0.77 ± 0.23	0.015*
PLR	Mean ± SD^a^	123.16 ± 64.47	91.89 ± 45.85	0.542
Claudin-5 (ng/L)	Median (IQR)^b^	30.94 (4-137)	44.12 (4–223.3)	0.680

^a^ independent sample t test

^b^ Mann Whitney test

*: Significant difference comparing between both groups at P value < 0.05

WBCs; White blood cells, NLR: Neutrophil/Lymphocyte ratio and PLR: Platelet/Lymphocyte ratio, SD; standard deviation and IQR; interquartile range

**Table 3:** Correlation analysis between total Conner’s scale and subscales with inflammatory markers and Claudin-5

Variable	Conner’s		Hyperactivity		Impulsivity		Inattention	
	r	p	r	p	r	p	r	p
WBCs	0.297	0.094	0.013	0.942	0.094	0.603	0.346	0.049*
Lymphocytes	0.400	0.021*	0.139	0.441	0.193	0.283	0.485	0.004*
Neutrophils	0.028	0.876	-0.176	0.326	-0.108	0.55	0.028	0.877
Platelets	-0.25	0.889	-0.089	0.623	-0.078	0.665	0.081	0.654
NLR	-0.350	0.046*	-0.260	0.144	-0.363	0.038*	-0.339	0.054
PLR	-0.459	0.007*	-0.162	0.366	-0.211	0.240	-0.457	0.008*
Claudin-5	-0.203	0.258	-0.196	0.274	-0.157	0.383	-0.298	0.093

Analyzed by Pearson correlation test

*Indicates P-value < 0.05 (two-tailed)

WBCs; White blood cells, NLR: Neutrophil/Lymphocyte ratio and PLR: Platelet/Lymphocyte ratio

**Table 4:** Linear regression analysis for prediction of Claudin-5 serum levels

Variables	Claudin-5	
	Standardized coefficient B	*P* value
Age	-0.182	0.682
Gender	-0.164	0.213
GA	-0.071	0.594
weight	-1.118	0.139
Height	1.217	0.071
BMI	-0.215	0.367
WBCs	1.531	0.077
Lymphocytes	0.503	0.154
Neutrophils	1.123	0.023*
Platelets	-0.266	0.380
NLR	-0.148	0.558
PLR	0.460	0.141

*Indicates P-value < 0.05

BMI; Body mass index, GA; Gestational age, WBCs; White blood cells, NLR: Neutrophil/Lymphocyte ratio and PLR: Platelet/Lymphocyte ratio

## Discussion

To the best of our understanding, this is one of the few research investigations to correlate impaired BBB integrity with peripheral inflammation in ADHD children. In this investigation, we discovered lower levels of serum Claudin-5 in ADHD children than in healthy controls, indicating that BBB integrity may be affected in ADHD children.

Claudin-5 is an essential part of endothelial tightly bound junctions (TJ) in the BBB, inhibiting harmful chemicals’ flow to the brain [23].

Numerous studies have investigated Claudin-5 and its role in mental illnesses; Ferahkaya et al. (2024) discovered that Claudin-5 levels were considerably lower in ADHD children compared to norms. Also, two recent investigations found lower claudin-5 levels among individuals with mood disorders and psychosis [11]. However, one other investigation on the link involving Claudin-5 and ADHD found that persons with ADHD had higher serum Claudin-5 levels than those in the control group [24]. The researchers attempted to argue that the elevated Claudin-5 levels found in the ADHD group could be attributed to an adaptive response against Claudin-5-related disturbance in BBB.

However, our results are more convincing as many studies show that decreased tight junction proteins are associated with mental illness, as in a study using experimental animals, rats subjected to prolonged stress showed decreased Claudin-5 levels and diminished function in TJs in vascular tissues. The researchers argued that a decline of function in TJs produces a rise in the permeation of the BBB, resulting in depression-like symptoms in rats [25].

The results of the current study indicated differences between children with ADHD and the control group regarding blood inflammatory cells: NLR (*p* = 0.015) and neutrophil count (*p* = 0.011), with higher values in the ADHD group; also, the lymphocyte counts were lower in ADHD children than control, yet the difference is still insignificant.

These results were consistent with Abdelsamieh et al., 2024, who discovered a statistically significant difference between those with ADHD and their control group in all of the blood inflammatory indicators (excluding platelet number) [26].

A different study conducted by Avcil and colleagues (2018) produced nearly identical results; the authors observed a statistically significant difference between the patients and control group in terms of NLR, PLR, neutrophil number, lymphocyte number, and number of platelets [16]. In a third investigation, the entire patient group had considerably higher NLR and neutrophils than controls and lower lymphocytes [27].

On investigating the association between peripheral markers of inflammation (WBCs, neutrophils, lymphocytes, NLR, and PLR) and the degree of severity of ADHD symptoms throughout the patient group. Our findings revealed that elevated lymphocyte levels are associated with an increase in ADHD symptoms, particularly total Conner’s scale, and inattention, whereas increased NLR and PLR play a protective role by decreasing ADHD symptom severity, particularly total Conner’s scale, impulsivity, and inattention. However, the previously mentioned studies didn’t find a significant relationship between inflammatory cells and symptoms severity. This may be because they stratified the patients into domain subtypes, which led to a decrease in the sample size that didn’t clarify any relationship if present.

ADHD symptoms. Many researches indirectly study the impact of lymphocytes on the severity of ADHD symptoms by measuring proinflammatory cytokines, which can easily enter the brain via a disrupted BBB, as our findings show. It was discovered that excessive amounts of pro-inflammatory cytokines can affect the plasticity of synapses and neurogenesis [28].

Accordingly, cytokines can alter cognitive processes such as response time and memory consolidation [29], which can be disrupted in ADHD. Increased expression of pro-inflammatory cytokines like Interferon-gamma and tumor necrosis factor alpha affects tryptophan biosynthesis. Tryptophan metabolites have been found to affect a variety of neurotransmitter networks, including dopaminergic conduction. Reduced levels of tryptophan and its metabolites have been linked to the extent of ADHD symptoms [30].

Proinflammatory cytokines may additionally trigger microglia. When active, microglia create additional pro-inflammatory cytokines, which increase microglial activation, producing an inflammatory advance that leads to neuroinflammation and, presumably, the pathogenesis of ADHD [31].

In continuing analysis of the interplay between inflammatory cells, Claudin-5 levels, and ADHD symptom severity, in our study, neutrophils show a positive association with Claudin-5 levels, which may act to protect the integrity of the BBB via an increase in the Claudin-5 expression in the TJs of the endothelium of the BBB. Elevated NLR in our patients is mostly caused by increased circulating neutrophil numbers, as there is a significant difference between patients and healthy controls.

It is known that stress inducement frequently leads to increased neutrophil counts in people [32]; furthermore, as opposed to other white blood cells, neutrophils have been demonstrated to raise most in mice that have experienced stress and keep on rising hours later, even as other white blood cell numbers begin to decline, showing the long-term effect of stress on neutrophil generation and discharging [33].

One systematic review found proof for stress-related brain changes that could either directly or indirectly lead to the emergence or worsening of diagnosed ADHD or ADHD symptoms. Heidt and associates explored the potential mechanism for enhanced neutrophil generation during stress and discovered that noradrenaline, a hormone linked to stress, was raised in the marrow cells of stressed rodents [32]. Noradrenaline binding to beta-3 adrenergic receptors in mesenchymal stem cells reduces cyclo-oxygenase 12 levels, which inhibits hematopoietic stem and progenitor cell growth and the migration of neutrophils. Thus, when exposed to prolonged stress, hematopoietic stem and progenitor cells proliferate and differentiate into neutrophils and other white blood cells, entering the circulation and raising NLR readings [32].

In our study, we postulate that elevated neutrophils have a repairing role in the BBB via up-regulation of Claudin-5. In support of our hypothesis, in 2019, Lund et al. observed that there is a link between neutrophil recovery following hematopoietic stem cell transplantation (HSCT) and early repair of the BBB (gadolinium resolution) in children with adrenoleukodystrophy by evaluating the pre-HSCT characteristics and 1-year post-HSCT outcomes in 66 boys undergoing marrow or cord blood HSCT [34].

In the same subject matter, Bansal et al. (2016) revealed promising findings in small pilot research employing autologous bone marrow aspirate concentrate administered intrathecally into 10 recruited autistic individuals, with a reduction in symptoms along 1 year of follow-up [35].

A small sample size and one-center study may act as possible limitations in the current study. Drug-treated children should be included in further research to compare NLR and Claudin-5 before and after treatment; also, the BBB integrity and neutrophil correlation should be confirmed by both laboratory and imaging studies in future studies, and the role of increasing neutrophil levels should be investigated thoroughly if it can be a modality for the treatment of severe cases of ADHD, also, the use of cytokines in future researches concerning inflammatory cells and Claudin-5 should be considered.

## Conclusion

BBB integrity is affected in ADHD children rather than healthy controls, as Claudin-5 levels were found to be significantly lower in ADHD children. Neutrophil count and neutrophil lymphocyte ratio (NLR) are significantly higher in cases than controls, and they are linked to decreased ADHD symptom severity, as increased neutrophils were shown to up-regulate Claudin-5 levels, while lymphocytes are responsible for symptom enhancement in ADHD children.

## Data Availability

No datasets were generated or analysed during the current study.

## Abbreviations

ADHD: attention deficit hyperactivity disorder
NLR: Neutrophil/lymphocyte ratio
PLR: Platelets/lymphocyte ratio
BBB: Blood brain barrier
HSCT: Heamatopoietic stem cell transplantation
